# PRKN‐Mediated Ubiquitin‐Proteasome Degradation of METTL3 Promotes Cellular Senescence

**DOI:** 10.1111/acel.70347

**Published:** 2025-12-29

**Authors:** Liping Chen, Canfeng Zhang, Yuanlong Ge, Haoxian Zhou, Shenglong Yang, Wenjing Wei, Qinghua Zhou, Kaizhen Xiao, Guangyu Huang, Xiaocui Li, Jia Wang, Jinping Zheng, Ronghe Gu, Zhenyu Ju, Shu Wu

**Affiliations:** ^1^ The Center for Medical Research The Fifth Affiliated Hospital of Guangxi Medical University Nanning Guangxi P. R. China; ^2^ Guangxi Key Laboratory of Intelligent Precision Medicine The First People's Hospital of Nanning Nanning Guangxi P. R. China; ^3^ Center for Translational Medicine, the First Affiliated Hospital Sun Yat‐Sen University Guangzhou Guangdong P. R. China; ^4^ Key Laboratory of Regenerative Medicine of Ministry of Education, Institute of Aging and Regenerative Medicine, Department of Developmental & Regenerative Medicine, College of Life Science and Technology Jinan University Guangzhou Guangdong P. R. China; ^5^ Department of Cardiology Guangdong Provincial Cardiovascular Institute, Guangdong Provincial People's Hospital, Guangdong Academy of Medical Sciences Guangzhou Guangdong P. R. China; ^6^ Key Laboratory of Regenerative Medicine of Ministry of Education, Institute of Aging and Regenerative Medicine and the First Affiliated Hospital Jinan University Guangzhou Guangdong P. R. China; ^7^ Department of Orthopedic Surgery The Fifth Affiliated Hospital of Guangxi Medical University Nanning Guangxi P. R. China; ^8^ Department of Cell Biology and Genetics, School of Pre‐Clinical Medicine Guangxi Medical University Nanning Guangxi P. R. China; ^9^ Shanxi Key Laboratory of Aging Mechanism Research and Translational Applications Changzhi Medical College Changzhi Shanxi P. R. China

**Keywords:** m6A, METTL3, PRKN, senescence, telomere

## Abstract

N6‐methyladenosine (m6A) methylation, a dynamic and reversible modification of eukaryotic mRNAs, plays critical roles in diverse cellular processes. Although METTL3‐mediated m6A deposition has been implicated in cellular senescence, the mechanisms controlling METTL3 stability and activity during senescence remain poorly defined. Here, we demonstrate that both m6A levels and METTL3 protein abundance are significantly reduced in replication‐induced and stress‐induced senescence models. *METTL3* depletion promotes senescence by inducing telomere dysfunction via diminished expression of shelterin components TRF2 and POT1. Mechanistically, we identify PRKN (Parkin) as a senescence‐associated E3 ubiquitin ligase that promotes METTL3 proteasomal degradation through K48‐linked polyubiquitination at lysine 164. Genetic *PRKN* inhibition in pre‐senescent cells rescues METTL3 expression, restores TRF2/POT1 levels, reduces telomere dysfunction‐induced foci (TIFs), and attenuates senescence‐associated β‐galactosidase (SA‐β‐gal) activity. Crucially, PRKN overexpression accelerates telomere dysfunction and senescence in wild‐type METTL3‐expressing cells but not in cells expressing the ubiquitination‐resistant K164R METTL3 mutant. Our findings establish METTL3 ubiquitination as a pivotal regulator of telomere integrity and senescence progression, unveiling a therapeutic target for age‐related pathologies.

## Graphical abstract

Schematic diagram of model for PRKN mediate METTL3 proteasome degradation promoting cellular senescence. The left panel shows a high abundance of METTL3 expression in young cells. METTL3 mediates m6A modification on TRF2 and POT1 mRNA, leading to their high expression and maintaining telomere stability. The right panel demonstrates that in senescent cells, PRKN is upregulated facilitating METTL3 degradation through the ubiquitin‐proteasome pathway. This reduction in METTL3 results in decreased of TRF2 and POT1 levels, inducing telomere dysfunction and accelerating cell senescence. (Created with BioGDP.com).

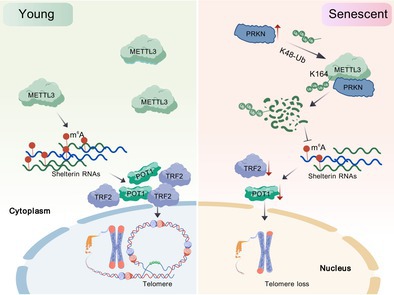

## 1 Introduction

N6‐methyladenosine (m6A), the most common modification in eukaryotic mRNA, serves as a central regulator of post‐transcriptional gene expression by modulating RNA splicing, stability, and translation efficiency (Roundtree et al. 2017). This dynamic modification is orchestrated by three interdependent molecular systems: writers (e.g., METTL3‐METTL14‐WTAP methyltransferase complexes), erasers (e.g., FTO and ALKBH5 demethylases), and readers (e.g., YTHDF, YTHDC, and IGF2BP families), which decode m6A‐mediated signaling pathways (Meyer and Jaffrey 2014). Mounting evidence implicates m6A dysregulation in cellular senescence and age‐related pathologies. Notably, m6A deposition patterns directly shape the expression of senescence‐associated genes including MIS12, HSPA1A, NPNT, and GPX4, positioning this epitranscriptomic mechanism as a potential modulator of aging dynamics (Jing et al. 2024; Wang et al. 2024; Wu et al. 2023, 2020).

Cellular senescence, defined as an irreversible cell cycle arrest state, arises through two distinct pathways: replicative senescence driven by telomere attrition and stress‐induced premature senescence triggered by genomic insults such as DNA damage or oxidative stress (d'Adda di Fagagna 2008). While transient senescence acts as a tumor‐suppressive mechanism, pathological accumulation of senescent cells promotes tissue dysfunction and age‐related disease progression. Our previous work established METTL3‐dependent m6A methylation as an essential guardian of telomere stability in malignant cells (Chen et al. 2022; Lee et al. 2021), revealing its fundamental role in chromosomal homeostasis. Nevertheless, critical knowledge gaps persist: (i) Does METTL3‐mediated m6A methylation exert comparable telomere‐protective functions in non‐transformed somatic cells? (ii) Through what molecular logic might m6A modifications coordinate telomere integrity with senescence regulation?

Intriguingly, METTL3 expression is consistently downregulated in aging tissues and age‐related disease models (Arcidiacono et al. 2020; Min et al. 2018) yet the mechanisms driving this decline remain enigmatic. Post‐translational modifications (PTMs), particularly ubiquitination, represent a potent regulatory layer controlling protein stability and activity. The ubiquitination cascade—orchestrated by E1 activating enzymes, E2 conjugating enzymes, and E3 ligases—dictates substrate fates through two canonical pathways: proteasomal degradation primarily mediated by K48‐linked polyubiquitin chains, or non‐degradative regulation, as exemplified by signaling modulation via K63‐linked ubiquitin chains (Hershko and Ciechanover 1998). While METTL3 regulation in cancer has been partially characterized, its PTM‐driven control during senescence remains unexplored.

Here, we delineate a ubiquitin‐mediated degradative axis controlling METTL3 homeostasis in senescence. We identify PRKN (Parkin) as the E3 ligase that catalyzes METTL3 K48‐linked polyubiquitination at lysine 164, triggering its proteasomal degradation. This mechanism functionally couples METTL3 instability to telomere dysfunction and senescence progression. Our findings establish the molecular mechanism underlying METTL3 downregulation during aging and identify a targetable node for therapeutic intervention in senescence‐associated pathologies.

## 2 Results

### 2.1 METTL3 Protein Levels but Not RNA Levels Are Decreased in Senescent Cells

**FIGURE 1:**
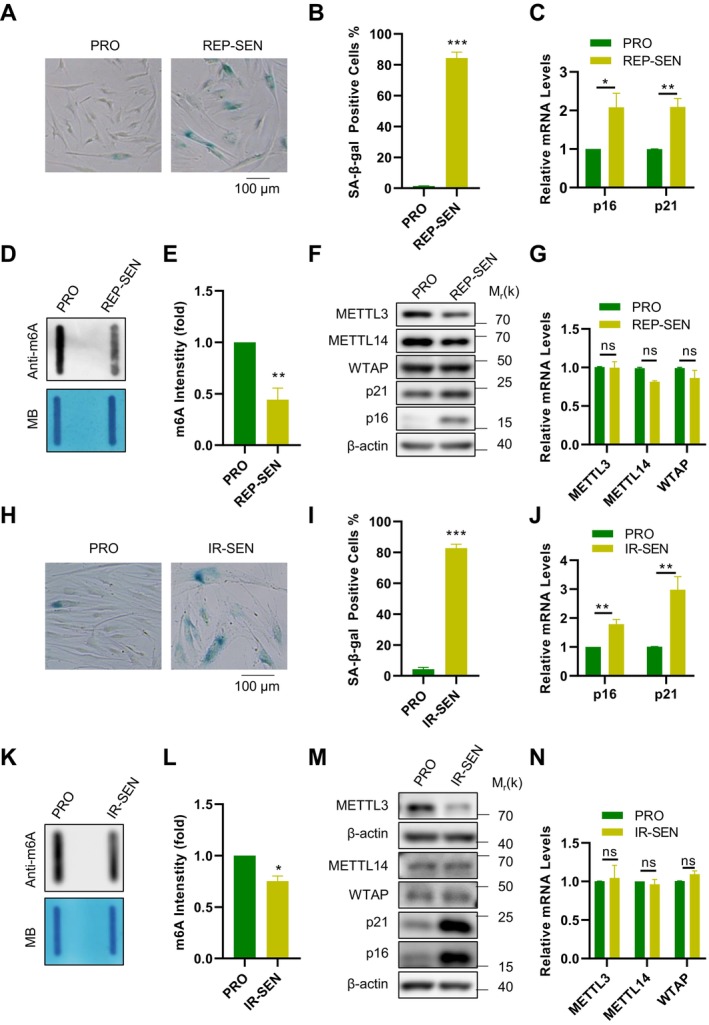
METTL3 protein levels decreased in senescent cells. (A) SA‐β‐gal staining was performed in proliferative (PRO) and replicative senescent cells (REP ‐ SEN). Scale bar: 100 μm. (B) Quantification of the staining results shown in panel (A). (C) mRNA levels of p16 and p21 were assessed using RT‐qPCR. (D) Detection of total RNA m6A signals in proliferative and replicative senescent BJ cells by Slot Blot. Methylene blue (MB) staining was used as RNA loading control. (E) Quantification of the m6A signals shown in panel (D). (F) Proteins expression levels of METTL3, METTL14 and WTAP in proliferative and replicative senescent BJ cells were determined by Western Blot. (G) mRNA levels of METTL3, METTL14 and WTAP in proliferative and replicative senescent BJ cells were assessed using RT‐qPCR. (H) SA‐β‐gal staining was performed in cells subjected to control or 10 Gy X‐ray irradiation. Scale bar: 100 μm. (I) Quantification of the staining results shown in panel (H). (J) mRNA levels of p16 and p21 were detected by RT‐qPCR. (K) Detection of total RNA m6A signals in proliferative (PRO) and X‐ray irradiation BJ cells. Methylene blue (MB) staining was used as RNA loading control. (L) Quantification of the m6A signals shown in panel (K). (M) Proteins expression levels of METTL3, METTL14 and WTAP proteins in proliferative (PRO) and X‐ray irradiation BJ cells were determined by Western Blot. (N) mRNA levels of METTL3, METTL14 and WTAP in proliferative (PRO) and X‐ray irradiation BJ cells were assessed using RT‐qPCR. Data represent mean ± SEM of three or more biological replicates. Statistical significance was determined by two‐tailed unpaired t‐tests (Student's method for equal variances, Welch's correction if applicable): *p < 0.05, **p < 0.01, ***p < 0.001.

To investigate whether m6A methyltransferases drive senescence‐associated epigenetic remodeling, we compared proliferating and replicative senescent BJ fibroblasts and quantified global m6A alongside core methyltransferases. Cellular senescence was confirmed by SA‐β‐gal staining (Figure 1A,B) and increased expression of the senescence markers p16 (CDKN2A‐encoded protein) and p21 (CDKN1A‐encoded protein) (Figure 1C). Quantitative Slot Blot analysis revealed a significant reduction in global m6A levels in senescent cells compared to proliferating controls (Figure 1D,E), establishing senescence‐associated m6A hypomethylation.

Given this hypomethylation phenotype, we profiled core m6A methyltransferases. Western Blot analysis showed decreased protein abundance of METTL3 and METTL14 in replicative senescent cells (Figure 1F), whereas RT‐qPCR indicated unchanged transcript levels (Figure 1G). Notably, WTAP protein remained stable (Figure 1F), consistent with its unaltered mRNA expression (Figure 1G).

To determine whether this regulatory paradigm extends beyond replicative senescence, we established an irradiation‐induced senescence model (10 Gy X‐ray). Consistent with replicative senescence, irradiated cells exhibited comparable m6A reduction (Figure 1H–L) and METTL3 protein depletion without transcriptional alterations (Figure 1M,N). Critically, WTAP protein and mRNA levels remained unaltered in irradiated cells (Figure 1M,N), while METTL14 protein showed no significant reduction in this context (Figure 1M,N). These convergent dynamics—reduced METTL3 protein without transcriptional changes in both senescence models—implicate post‐translational mechanisms in its regulation, positioning METTL3 as a conserved senescence‐associated factor with post‐translational vulnerability.

### 2.2 PRKN Expression Is Upregulated in Senescent Cells and Mediates the Ubiquitination of METTL3

**FIGURE 2:**
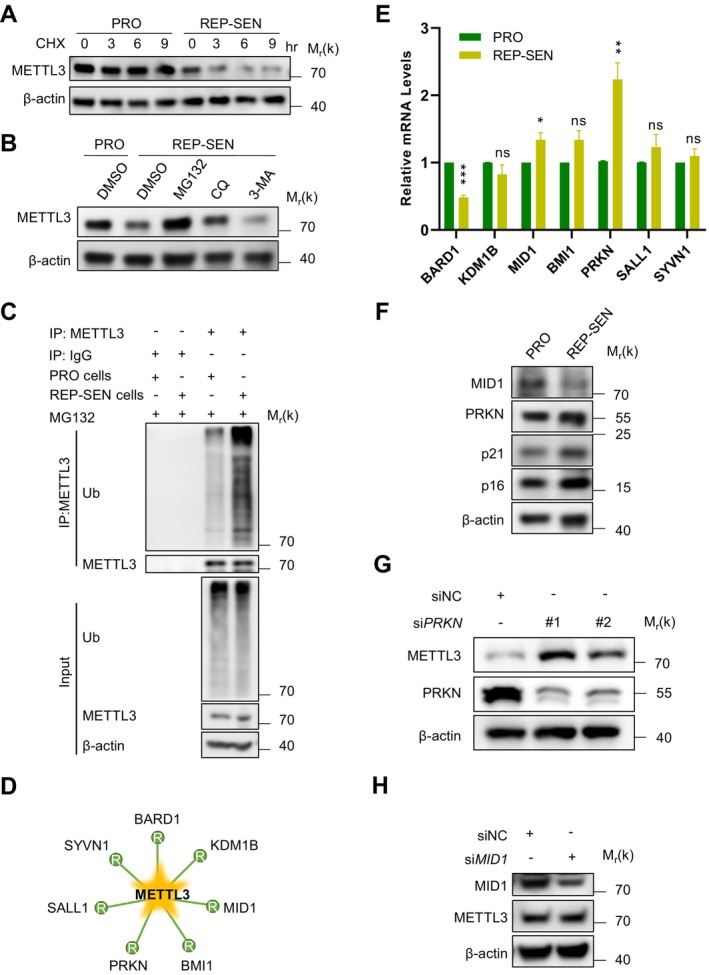
PRKN mediates ubiquitination modification of METTL3 in senescent cells. (A) Western Blot analysis of METTL3 protein levels in proliferative and replicative senescent cells treated with cycloheximide (CHX, 100 mg/mL) at various time points. (B) Western Blot analysis of METTL3 protein levels in proliferative and replicative senescent BJ cells treated with MG132 (10 μM), CQ (100 μM) or 3‐MA (10 mM) for 9 h. The cell lysates were analyzed by Immunoblot. (C) Co‐immunoprecipitation and Western Blot analysis of extracts from proliferative and replicative senescent BJ cells treated with MG132 (10 μM) for 9 h. (D) Potential E3 ubiquitin ligases for METTL3 as predicted by UbiBrowser 2.0 database. (E) mRNA levels of potential E3 ubiquitin ligases were measured in proliferating and senescent cells were assessed using RT‐qPCR. (F) protein expression levels of PRKN and MID1 in proliferating and senescent cells were measured by Western Blot analysis. (G) Western Blot analysis to determine METTL3 expression and PRKN knockdown efficiency in BJ cells transfected with PRKN or control siRNA. (H) METTL3 protein expression and MID1 knockdown efficiency were measured by Western Blot analysis following transfection with MID1 or control siRNA. Data represent mean ± SEM of three or more biological replicates. Statistical significance was assessed using two‐tailed unpaired Student's t‐tests (for data with equal variances) or Welch's corrected t‐tests (for unequal variances): *p < 0.05, **p < 0.01, ***p < 0.001.

Given the conserved discordance between METTL3 protein depletion and stable mRNA levels across senescence models (Figure 1F,G,M,N), we hypothesized accelerated protein turnover during senescence. To investigate this, cycloheximide (CHX) chase assays in replicative senescent cells revealed accelerated METTL3 degradation compared to proliferating controls (Figure 2A).

To further explore the specific post‐translational mechanisms responsible for METTL3 degradation, we assessed its stability in the presence of various inhibitors. Our data indicated that METTL3 degradation was inhibited by the proteasome inhibitor MG132, whereas no significant effects were observed with the lysosome inhibitor chloroquine (CQ) or the autophagy inhibitor 3‐MA (Figure 2B). Since ubiquitination is a key signal for proteasomal degradation, we next examined whether the ubiquitination of METTL3 was altered in senescent cells. The results revealed a significant increase in METTL3 ubiquitination in senescent cells (Figure 2C).

To identify the responsible E3 ligase, we performed UbiBrowser 2.0 analysis (Wang et al. 2022), which revealed seven candidates: BARD1, KDM1B, MID1, BMI1, PRKN (Parkin), SALL1 and SYVN1 (Figure 2D). To determine which candidate E3 ligases mediate METTL3 ubiquitination, qPCR analysis of these seven candidate E3 ligases showed that only PRKN and MID1 exhibited upregulated mRNA levels in senescent cells (Figure 2E). Subsequent Western blot analysis demonstrated exclusively PRKN protein elevation under senescence conditions (Figure 2F). Functional validation via siRNA‐mediated knockdown revealed that *PRKN* ablation increased METTL3 protein levels (Figure 2G), whereas *MID1* silencing had no effect (Figure 2H). This differential regulation establishes PRKN as the principal E3 ligase governing METTL3 stability during cellular senescence.

### 2.3 PRKN Directly Mediates the Proteasomal Degradation of METTL3 via k48‐Linked Ubiquitination

**FIGURE 3:**
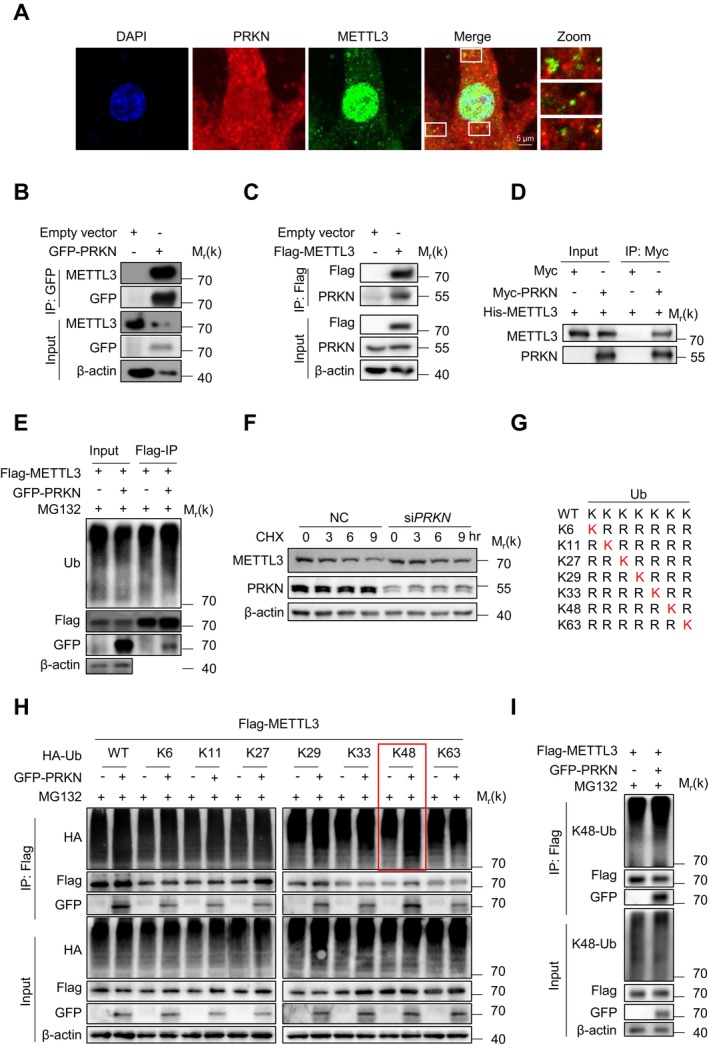
PRKN mediates the proteasomal degradation of METTL3 via k48 linkage. (A) Immunofluorescence analysis of PRKN and METTL3 in BJ cells using anti‐PRKN antibody followed by Alexa Fluor 594‐conjugated secondary antibody (red) and anti‐METTL3 antibody followed by Alexa Fluor 488‐conjugated secondary antibody (green). Scale bar: 5 μm. (B) Immunoprecipitation followed by Western Blot analysis was performed on protein extracts from HEK293T cells transfected with GFP‐PRKN or empty vector using anti‐GFP beads. (C) Immunoprecipitation followed by Western Blot analysis was performed on protein extracts from HEK293T cells transfected with Flag‐METTL3 or empty vector using anti‐Flag beads. (D) PRKN binds METTL3 directly. In vitro pull‐down assay using exogenously purified Myc‐PRKN protein and His‐METTL3 protein. Myc peptide was added in the control group (E) Co‐immunoprecipitation and Western Blot analysis of extracts from HEK293T cells transfected with Flag METTL3, together with the empty vector or GFP‐PRKN and treated with MG132 (10 μM) for 9 h using anti‐Flag beads and with anti‐ubiquitin (Ub) antibody for detection. (F) Immunoblot analysis of METTL3 protein levels in HEK293T cells transfected with PRKN or control siRNA, followed by treated with cycloheximide (CHX, 100 mg/mL) at the indicated time points. (G) Schematic diagram of Ub mutants. (H) Co‐immunoprecipitation and Western Blot analysis of extracts from HEK293T cells transfected with Flag‐METTL3 and HA‐tagged Ub mutants (and controls), together with the empty vector or GFP‐PRKN, treated with MG132 (10 μM) for 9 h. Immunoprecipitated with anti‐Flag beads and analyzed by immunoblot with anti‐HA antibody. (I) Co‐immunoprecipitation and Western Blot analysis of extracts from HEK293T cells transfected with Flag‐METTL3, together with the empty vector or GFP‐PRKN and treated with MG132 (10 μM) for 9 h using anti‐Flag beads and immunoblotted with anti‐K48‐ubiquitin (K48‐Ub) antibody.

To elucidate the molecular mechanisms by which PRKN mediates METTL3 degradation, we first investigated their interaction. Immunofluorescence (IF) analysis in BJ fibroblasts revealed evident PRKN‐METTL3 colocalization (Figure 3A). Co‐immunoprecipitation (co‐IP) in HEK293T cells further confirmed a robust physical interaction between PRKN and METTL3 (Figure 3B,C). To establish direct binding, bacterially expressed and purified His‐METTL3 and Myc‐PRKN proteins were purified and incubated in vitro. This reconstitution assay demonstrated that METTL3 directly binds PRKN (Figure 3D).

Additionally, we analyzed the effect of PRKN on METTL3 ubiquitination and found that PRKN overexpression significantly enhanced the poly‐ubiquitination of METTL3 (Figure 3E). To assess whether PRKN influences the stability of METTL3, a cycloheximide (CHX) chase assay was performed. The results revealed that the depletion of *PRKN* delayed the degradation of METTL3 (Figure 3F). We further identified the specific type of ubiquitin chain added by PRKN using wild‐type (WT) ubiquitin and various mutants (K6‐, K11‐, K27‐, K29‐, K33‐, K48‐, and K63‐only) (Figure 3G). The K48‐only mutant, like WT ubiquitin, significantly increased PRKN‐induced METTL3 ubiquitination (Figure 3H). Moreover, overexpression of PRKN enhanced the K48‐linked ubiquitination of METTL3 in HEK293T cells (Figure 3I). These results demonstrate that PRKN catalyzes K48‐linked polyubiquitination of METTL3, targeting it for proteasomal degradation.

### 2.4 PRKN Mediates the Proteasomal Degradation of METTL3 by Adding Poly‐Ubiquitin Chains at K164

**FIGURE 4:**
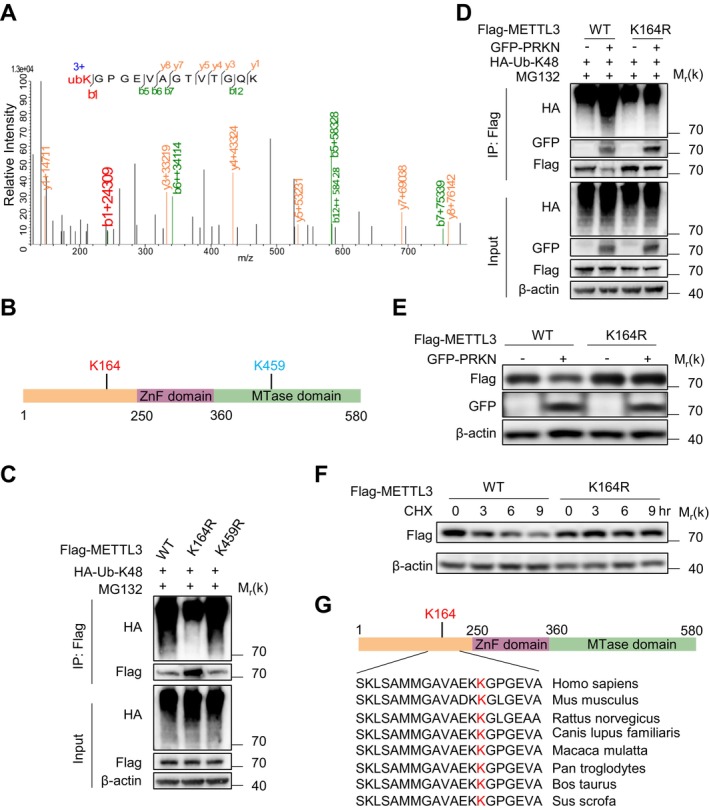
PRKN mediates the proteasomal degradation of METTL3 by adding poly‐ubiquitin chains at K164. (A) K164 ubiquitination of METTL3 in HEK293T cells co‐transfected with Flag‐METTL3 and GFP‐PRKN was assessed by mass spectrometry. (B) Schematic representation of METTL3 domains showing potential ubiquitination sites mediated by PRKN. (C) Co‐immunoprecipitation and Western Blot analysis of HEK293T cells transfected with wild type (WT) Flag METTL3 or its mutants, together with HA‐K48‐Ub, and treated with MG132 (10 μM) for 9 h. (D) Co‐immunoprecipitation and Western Blot analysis of HEK293T cells co‐transfected with wild type (WT) Flag‐METTL3 or its K164R mutant, together with empty vector or GFP‐PRKN and HA‐K48‐Ub, followed by treatment with MG132 (10 μM, 9 h). (E) Western Blot analysis of protein extracts from HEK293T cells transfected with WT Flag‐METTL3 or its K164R mutant, along with either empty vector or GFP‐PRKN. (F) Western Blot analysis of protein extracts from HEK293T cells transfected with WT Flag‐METTL3 or its K164R mutant and treated with cycloheximide CHX (100 mg/mL) for the indicated time points. (G) Sequence alignment of conserved lysine residues in METTL3‐K164 and homologous regions in METTL3 from various species. All data are measured more than three independent experiments.

To identify the lysine residues on METTL3 targeted for K48‐linked ubiquitination, we purified Flag‐tagged METTL3 and performed multidimensional liquid chromatography coupled with tandem mass spectrometry (2D‐LC–MS/MS) analysis (Figure 4A). Combined with predictive modeling, this approach identified two candidate ubiquitination sites: K164 and K459 (Figure 4B). To validate these sites, we generated Flag‐METTL3 mutants containing single lysine‐to‐arginine substitutions (K164R and K459R) and assessed their ubiquitination status. Ubiquitination assays revealed that the K164R mutant exhibited significantly reduced K48‐linked ubiquitination, whereas the K459R mutation showed no effect (Figure 4C).

Critically, PRKN failed to mediate K48‐linked ubiquitination of the METTL3 K164R mutant (Figure 4D), establishing K164 as essential for PRKN‐dependent ubiquitination. To determine the functional consequence of this modification, we compared protein stability between wild‐type (WT) METTL3 and the K164R mutant. Unlike WT METTL3, the K164R mutant resisted PRKN‐mediated degradation (Figure 4E). Consistently, cycloheximide (CHX) chase assays demonstrated delayed degradation kinetics of the K164R mutant compared to WT METTL3 (Figure 4F). Comparative analysis of the primary amino acid sequences of METTL3 homologs across species revealed a high conservation of K164 among mammals (Figure 4G). These data establish that PRKN targets K164 for K48‐linked ubiquitination to drive proteasomal degradation of METTL3.

### 2.5 METTL3 Deficiency Promotes Telomere Dysfunction‐Induced Cellular Senescence

**FIGURE 5:**
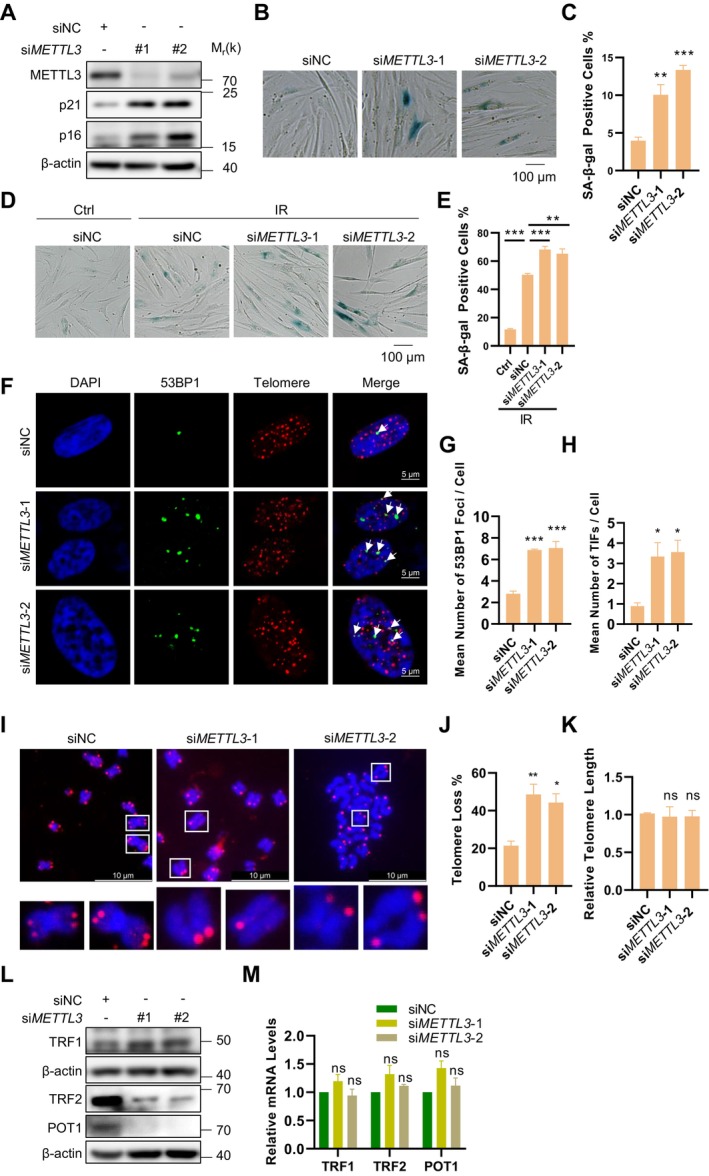
METTL3 deficiency promotes telomere dysfunction‐induced cellular senescence. (A) Western Blot detected the protein levels of p16/p21 and METTL3 knockdown efficiency in BJ cells following transduction with METTL3 and control siRNA. (B) SA‐β‐gal staining was performed on siMETTL3 BJ cells or control BJ cells. Scale bar: 100 μm. (C) Quantification of SA‐β‐gal staining in panel (B). (D) SA‐β‐gal staining was performed on siMETTL3 BJ cells or control BJ cells after X‐ray irradiation at 10 Gy. Scale bar: 100 μm. (E) Quantification of SA‐β‐gal staining in panel (D). (F) 53BP1 was detected by anti‐53BP1 antibody followed by Alexa Fluor 488‐conjugated secondary antibody (green) and telomeres were detected by telomere‐specific G probe (red) in BJ cells transfected with METTL3 and control siRNA. Scale bar: 5 μm. (G) Quantification of panel (F). The mean numbers of 53BP1 foci per cell were counted. More than 100 cells were counted in each sample for each independent experiment. (H) Quantification of panel (F). The mean number of 53BP1 foci co‐localized with telomeres was counted. (I) Fluorescence in situ hybridization (FISH) detection of telomeres on metaphase spreads of METTL3 depleted BJ cells. Scale bar: 10 μm. (J) Quantification of panel (I). The percentages of chromosomes with one or more telomere‐free ends were calculated. (K) Telomere length was measured by qPCR in BJ cells following transduction METTL3 and control siRNA for 2 weeks. (L) Proteins expression levels of TRF1, TRF2 and POT1 detected by immunoblotting. (M) mRNA levels of TRF1, TRF2 and POT1 were assessed by RT‐qPCR in BJ cells. Data represent mean ± SEM of three or more biological replicates. Statistical significance was determined by one‐way ANOVA with Holm‐Sidak's post hoc multiple comparisons test: *p < 0.05, **p < 0.01, ***p < 0.001.

To evaluate the functional impact of *METTL3* deficiency on cellular senescence, we first performed Western Blot analysis to detect the protein levels of the senescence markers p16 and p21 following *METTL3* depletion. The results demonstrated that loss of *METTL3* led to a significant upregulation of both p16 and p21 (Figure 5A). Subsequently, SA‐β‐gal staining assays were performed on both replicative and stress‐induced senescent cells, revealing that *METTL3* deficiency resulted in a marked increase in the number of SA‐β‐gal positive cells (Figure 5B–E).

Given that telomere attrition triggers senescence and prior evidence implicating METTL3‐mediated m6A modification in telomere homeostasis (Chen et al. 2022), we hypothesized that METTL3 may regulate cellular senescence through its effects on telomere homeostasis. Immunofluorescence‐Fluorescence in situ hybridization (IF‐FISH) analysis demonstrated that *METTL3* knockdown led to a significant increase in 53BP1 foci, with most of these foci colocalizing with telomeres in fibroblasts (Figure 5F–H). Furthermore, we observed that *METTL3* deletion led to telomere uncapping but had no effect on telomere length (Figure 5I–K). These findings suggest that METTL3 plays a critical role in maintaining telomere structure and stability but is not directly involved in telomere lengthening.

Given the established association between shelterin complex dysfunction and telomere‐driven genomic instability (Raghuram and Mishra 2014), we performed systematic analysis of its core constituents: (i) TRF1 and TRF2, the double‐stranded DNA‐binding proteins that organize telomere loop architecture; (ii) POT1, the single‐stranded telomeric DNA‐binding protein critical for G‐overhang protection. RT‐qPCR and Western Blot analysis revealed a selective depletion of TRF2 and POT1 at the protein level in METTL3‐deficient cells, while their mRNA levels remained unchanged (Figure 5L,M). This observation implies that METTL3 likely regulates the translational efficiency or post‐translational stability of these shelterin components, rather than influencing their transcriptional output.

### 2.6 PRKN Facilitates Cellular Senescence via METTL3 Degradation

**FIGURE 6:**
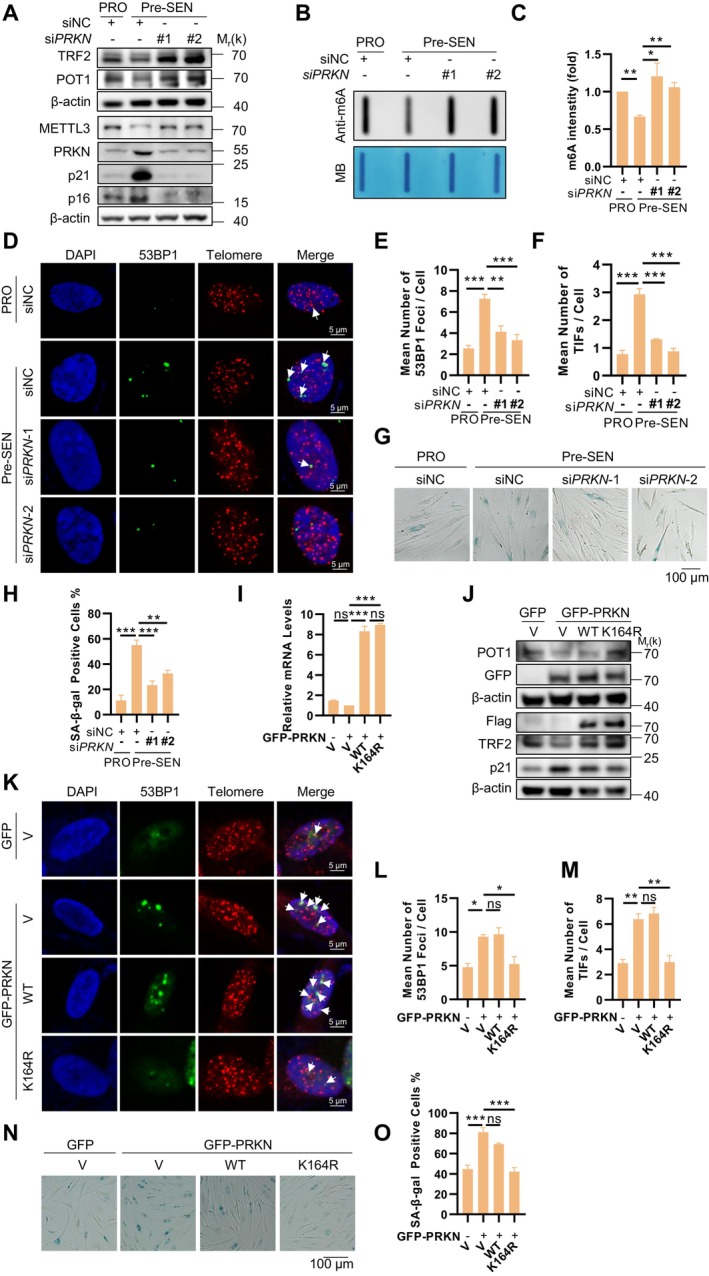
PRKN regulates METTL3 degradation‐induced cellular senescence. (A) Western Blot analysis of METTL3, p16 and p21, TRF2, POT1 protein levels and PRKN knockdown efficiency in proliferative and pre‐senescent BJ cell transfected with PRKN and control siRNA. (B) Detection of total RNA m6A levels in proliferative and pre‐senescent BJ cells transfected with PRKN and control siRNA. Methylene blue (MB) staining was used as RNA loading control. (C) Quantification of the m6A signals shown in panel (B). (D) 53BP1 was detected by anti‐53BP1 antibody followed by Alexa Fluor 488‐conjugated secondary antibody (green) and telomeres were detected by telomere‐specific G probe (red) in BJ cells which transfected with PRKN and control siRNA. Scale bar: 5 μm. (E) Quantification of panel (D). The mean number of 53BP1 foci per cell was counted. More than 100 cells were counted in each sample for each independent experiment. (F) Quantification of panel (D). The mean numbers of 53BP1 foci co‐localized with telomeres was counted. (G) SA‐β‐gal staining was performed on siPRKN BJ cells or control BJ cells. Scale bar: 100 μm. (H) Quantification of SA‐β‐gal staining results from panel (G). (I) mRNA levels of METTL3 in BJ cells which transfected with WT Flag‐METTL3 or its K164R mutant, along with either empty vector or GFP‐PRKN. (J) Western Blot detected TRF2, POT1, Flag METTL3, GFP‐PRKN and p21 in BJ cells transfected with WT Flag‐METTL3 or its K164R mutant, along with either empty vector or GFP‐PRKN. (K) 53BP1 was detected by anti‐53BP1 antibody followed by Alexa Fluor633‐conjugated secondary antibody (pseudo‐green) and telomeres were detected by telomere‐specific G probe (red) in BJ cells which transfected with WT Flag METTL3 or its K164R mutant, along with either empty vector or GFP‐PRKN. Scale bar: 5 μm. (L) Quantification of panel (K). The mean number of 53BP1 foci per cell was counted. More than 100 cells were counted in each sample for each independent experiment. (M) Quantification of panel (K). The mean numbers of 53BP1 foci co‐localized with telomeres was counted. (N) SA‐β‐gal staining was performed on BJ cells transfected with WT Flag‐METTL3 or its K164R mutant, along with either empty vector or GFP‐PRKN. Scale bar: 100 μm. (O) Quantification of SA‐β‐gal staining results from panel (N). Data represent mean ± SEM of three or more biological replicates. Statistical significance was determined by one‐way ANOVA with Holm‐Sidak's post hoc multiple comparisons test: *p < 0.05, **p < 0.01, ***p < 0.001.

Building on our finding that PRKN promotes proteasomal degradation of METTL3 (Figure 4), we investigated its role in cellular senescence by knocking down *PRKN* in pre‐senescent cells. Western Blot analysis revealed that *PRKN* deficiency restored METTL3 protein levels, subsequently rescuing expression of shelterin components TRF2 and POT1 while reducing senescent markers p16 and p21 expression (Figure 6A). Consistent with METTL3 functional recovery, global m6A levels reverted to those observed in proliferating cells (Figure 6B,C).

To determine whether *PRKN* knockdown alleviates METTL3 loss‐induced telomere dysfunction and senescence, we performed IF‐FISH and SA‐β‐gal staining assays in *PRKN*‐deficient cells. As expected, *PRKN* deficiency mitigated telomere dysfunction, evidenced by a decrease in telomeric 53BP1 foci formation (Figure 6D–F), and subsequently delayed the onset of cellular senescence, as demonstrated by a reduction in the proportion of SA‐β‐gal‐positive cells (Figure 6G,H). These results suggest that PRKN may regulate cellular senescence via the METTL3‐m6A‐telomere axis.

To further confirm that PRKN regulates cellular senescence by mediating proteasomal degradation of METTL3, we analyzed BJ cells overexpressing either wild‐type METTL3 (METTL3‐WT) or the ubiquitination‐resistant K164R mutant (METTL3‐K164R) following PRKN overexpression. Given that METTL3 mRNA levels remained unchanged (Figure 6I), PRKN overexpression specifically reduced METTL3‐WT but not METTL3‐K164R protein levels (Figure 6J). Concurrently, PRKN overexpression diminished TRF2/POT1 expression, while elevating p21 levels—effects rescued by METTL3‐K164R but not METTL3‐WT overexpression (Figure 6J). Consistent with this, PRKN overexpression promoted both total and telomeric 53BP1 foci formation, which were significantly attenuated in METTL3‐K164R‐ but not METTL3‐WT‐expressing cells (Figure 6K–M). Correspondingly, PRKN overexpression significantly increased the proportion of SA‐β‐gal‐positive cells in METTL3‐WT‐expressing cells, while METTL3‐K164R mutant cells exhibited resistance to this senescence acceleration (Figure 6N,O). Collectively, these findings demonstrate that PRKN‐mediated proteasomal degradation of METTL3 drives m6A‐dependent cellular senescence regulation.

Building on established evidence of accelerated cellular senescence in pulmonary systems—where senescent cells accumulate disproportionately during aging (Munoz‐Espin and Serrano 2014; Ovadya et al. 2018; Wang et al. 2009) and directly contribute to idiopathic pulmonary fibrosis (IPF) pathogenesis (Alvarez et al. 2017; Schafer et al. 2017; Zhang et al. 2024). To investigate PRKN's role in physiological aging and age‐related diseases, we quantified PRKN protein levels in lung tissues from young controls, aged mice, and mice with IPF. PRKN expression was significant upregulation in lung tissues from both aged mice (Figure S1A–D) and IPF models (Figure S1E–H). This convergent elevation of PRKN expression in two distinct but senescence‐exacerbated conditions reinforce its potential mechanistic role in pulmonary aging and fibrotic disease progression.

## 3 Discussion

Previous studies have established that METTL3‐mediated m6A modification regulates diverse cellular processes, including transcriptional control, RNA stability, and translational efficiency (Liu et al. 2020). Notably, several established METTL3‐regulated targets—including the cell proliferation regulator MIS12 (Wu et al. 2020), stress response mediators HSPA1A (Wang et al. 2024) and the ferroptosis‐suppressing antioxidant enzyme GPX4 (Jing et al. 2024); as well as the basement membrane adhesion molecule NPNT (Wu et al. 2023)—have been functionally associated with cellular senescence. This implicates METTL3 in regulating senescence via several distinct pathways, presenting a valuable direction for investigation. However, despite observed alterations in METTL3 expression and m6A deposition during senescence, the underlying molecular mechanisms remain poorly defined. Our study elucidates the mechanistic role of METTL3‐mediated m6A methylation in directly orchestrating telomere dysfunction and driving cellular senescence. Specifically, METTL3 deficiency reduces TRF2 and POT1 protein abundance, thereby promoting telomere dysfunction‐induced foci (TIF) formation and senescence initiation. Furthermore, we identify the E3 ubiquitin ligase PRKN (Parkin) as the driver of K48‐linked ubiquitination at METTL3 lysine 164, triggering its proteasomal degradation. Collectively, these findings establish the PRKN‐METTL3 axis as a critical regulator of telomere‐associated senescence through m6A dependent modulation.

Post‐translational modifications (PTMs) of METTL3, including phosphorylation and SUMOylation, have been characterized predominantly in oncogenic contexts, where they influence its enzymatic activity, stability, and subcellular localization (Shi et al. 2019; Zhang et al. 2020). Phosphorylation enhances METTL3's catalytic function and promotes tumor cell proliferation, while SUMOylation regulates nuclear retention and stability (Du et al. 2018; Zhang et al. 2020). Additionally, ubiquitination at sites such as K480 and K459 has been reported to target METTL3 for proteasomal degradation in tumor cells (Liao et al. 2022; Mao et al. 2025; Yang et al. 2023). However, whether PTMs of METTL3 contribute to cellular senescence and aging remains unclear. We identify K164 ubiquitination as a senescence‐specific regulatory mechanism. The K164R mutation abrogates PRKN‐mediated degradation, telomere dysfunction and attenuates senescence, indicating this residue critically controls METTL3 stability during aging. This cell fate‐dependent PTM pattern suggests METTL3 acts as a molecular switch, with distinct modification “codes” directing functional outcomes in proliferation versus senescence.

Our findings elucidate how m6A modification influences cellular senescence: METTL3 degradation in senescent cells reduces TRF2/POT1, causing telomere dysfunction. This contrasts with our prior work showing METTL3 promotes telomeric R‐loop formation via m6A‐modified TERRA, facilitating homologous recombination (HR) in ALT‐positive cancer cells (Chen et al. 2022). In somatic cells with minimal telomerase activity and HR efficiency, however, METTL3 maintains shelterin integrity through translational/post‐translational mechanisms that prevent telomere uncapping. This functional dichotomy highlights how cellular context dictates METTL3's roles, potentially explaining its divergent effects in oncogenesis versus aging.

PRKN is intricately associated with aging and age‐related diseases through diverse mechanisms. It plays a critical role in protein degradation, mitochondrial maintenance, and cellular stress responses, all of which are crucial for neuronal health. Mutations in PRKN can disrupt these processes, contributing to the neurodegenerative changes observed in Parkinson's Disease (Wahabi et al. 2018). In chronic obstructive pulmonary disease (COPD), Jun Araya et al. have demonstrated that PRKN in bronchial epithelial cells regulates mitochondrial autophagy, thereby delaying cellular senescence (Araya et al. 2019; Ito et al. 2015). Conversely, Xin Jin et al. observed that in the oocytes of aging mice, PRKN is upregulated, inhibiting mitochondrial autophagy and leading to abnormal meiosis and reduced oocyte quality (Jin et al. 2022). These findings indicate that the role of PRKN in regulating senescence is specific to cell type. Our work reveals that PRKN is elevated in senescent fibroblasts, where it mediates METTL3 degradation via K48‐linked ubiquitination—revealing a non‐canonical role in senescence independent of mitophagy.

The implications of our findings are profound in enhancing understanding of cellular senescence and aging. Maintaining stable METTL3 levels is crucial for telomere maintenance and overall cellular health. By elucidating how PRKN targets METTL3 for degradation, we identify a new regulatory pathway in cellular aging that could be exploited for therapeutic purposes. Strategies aimed at modulating PRKN activity or protecting METTL3 from ubiquitination and degradation may offer promising avenues for mitigating senescence‐associated pathologies.

Several limitations of this study warrant consideration. Firstly, while we established PRKN‐mediated METTL3 degradation in cellular models of senescence, the physiological relevance of this pathway in aging tissues remains to be validated. Further studies utilizing progeroid mouse models or METTL3 K164R mutant knock‐in mice could test whether blocking ubiquitination event attenuates age‐related telomere attrition. Secondly, the upstream triggers of PRKN upregulation during senescence remain unidentified. Systematic profiling of senescence inducers coupled with PRKN promoter analysis could identify potential regulators, such as SASP‐derived cytokines or mitochondrial ROS. Thirdly, beyond K164 ubiquitination, METTL3 stability may be regulated by other PTMs. For instance, phosphorylation at S43/S45, as reported in cancer cells, might antagonize PRKN‐mediated degradation. Employing integrated multi‐omics approaches (e.g., phosphoproteomics and ubiquitinomics) during senescence induction would be crucial to mapping this complex regulatory network and understand how METTL3 activity and stability are coordinately controlled to influence telomere homeostasis.

## 4 Material and Methods

### 4.1 Cell Culture

BJ, and HEK293T cells were obtained from American Type Culture Collection (ATCC) (Manassas, VA). Cells were cultured at 37°C in a humidified atmosphere containing 5% CO2. Cells were grown in Dulbecco's Modified Eagle Medium (DMEM) (Hyclone) with 10% fetal bovine serum (FBS) (Gibico) and 100 U/mL penicillin/streptomycin. All cells were confirmed to be free of for mycoplasma contamination.

### 4.2 Plasmids and Transfection

The wild‐type METTL3 gene was amplified from HEK293T mRNA and cloned into pHAGE‐Flag vector. METTL3‐K164R and METTL3‐K459R were generated by site‐directed mutagenesis, introducing a lysine (K) to arginine (R) substitution at positions 164 and 459, respectively, based on the wild‐type METTL3 sequence. The wild‐type PRKN gene was synthesized from IGEbiology and cloned into pLVX‐AcGFP1‐N1 vector. For transfection, BJ cells were transfected using Lipofectamine 3000 (Invitrogen), and HEK293T cells were transfected using PEI (Yeasen) according to the manufacturer's recommended protocol. Chemically synthesized 21‐nucleotide siRNA duplexes were purchased from GenePharma and transfected using Lipofectamine RNAiMAX (Invitrogen) according to the manufacturer's instructions. The RNA oligonucleotides used in this study are as below: si*METTL3*‐1: 5′‐CUGCAAGUAUGUUCACUAUGAdTdT‐3′; si*METTL3*‐2: 5′‐GCACUUGGAUCUACGGAAUCCdTdT‐3′; si*PRKN*‐1: 5′‐GCUUAGACUGUUUCCACUUAUdTdT‐3′; si*PRKN*‐2: 5′‐GCCUUCUGCCGGGAAUGUAAAdTdT‐3′; si*MIDI*: 5′‐GUCGUUAGUCUGUGUAAUUdTdT‐3′.

### 4.3 Slot Blot

1 μg RNA was diluted to a concentration of 10 ng/μL in a buffer containing 6 × SSC and 7.4% methanal. The samples were heated at 65°C for 15 min to denature secondary structures, followed by immediate cooling on ice. Subsequently, RNA was loaded onto Hybond *N*+ membranes. After air drying, the membranes were crosslinked using a UV stratalinker at an energy setting of 1200 J/cm^2^, applied six times. Membranes were then blocked for 1 h at room temperature in a solution of 5% non‐fat milk dissolved in PBST (0.1% Tween‐20). Following blocking, the membranes were incubated overnight at 4°C with anti‐6 mA antibody (Synaptic Systems, 1: 1000). On the second day, the membranes were washed five times, each for 5 min, with PBST. They were then incubated with HRP‐conjugated goat anti‐rabbit IgG secondary antibody (1:2000, 7074S, Cell Signaling Technology) for 1 h at room temperature. The washing step with PBST was repeated five times for 5 min each. Finally, the membranes were treated with enhanced chemiluminescence (ECL) reagent and developed for visualization of the antibody signals.

### 4.4 Immunoprecipitation and Immunoblot Analysis

For immunoprecipitation, Cells were lysed with RIPA buffer (50 mM Tris pH 7.4, 0.25% sodium deoxycholic acid, 1% Triton, 150 mM NaCl, 1 mM EDTA), supplemented with protease inhibitor. whole‐cell lysates were incubated overnight with the appropriate antibodies, followed by incubation with Protein A/G beads (sc‐2003, SANTA CRUZ), or anti‐Flag beads (Sigma). The beads were then washed five times with RIPA buffer, and the bound immunoprecipitants were eluted with 2 × SDS loading buffer and resolved by SDS‐PAGE. The following antibodies were used for immunoblotting: anti‐METTL3 (1:2000, ab195352, Abcam), anti‐METTL14 (1:3000, 26158‐1‐AP, Proteintech), anti‐WTAP(1:5000, 10200‐1‐AP, Proteintech), anti‐PRKN (1:1000, 14060‐1AP), anti‐p16 (1:1000, 10883‐1‐AP, Proteintech), anti‐p21 (1:1000, 2947S, CST), anti‐TRF1 (1:500, T1948, Sigma‐Aldrich), anti‐TRF2 (1:1000 dilution, 66893‐1‐IG, Proteintech), anti‐POT1 (1:500 dilution, 10581‐1‐AP, Proteintech), HRP‐conjugated goat anti‐rabbit (1:2000, 7074S, Cell Signaling Technology) or horse anti‐mouse (1:2000, 7076S, Cell Signaling Technology).

### 4.5 In Vitro Pull‐Down Assay

Recombinant His‐METTL3 and Myc‐PRKN proteins (purchased from Targetmol) were incubated with Myc‐Beads in RIPA buffer (1% NP‐40, 0.25% sodium deoxycholate, 50 mmol/L Tris‐HCl (pH 7.4) and 150 mmol/L NaCl) at 4°C for 1 h. Myc peptides were used as a control. After incubation, beads were washed four times with RIPA buffer. The bound proteins were analyzed by Western Blot using anti‐METTL3 (1:2000, ab195352, Abcam) and anti‐PRKN (1:1000, 14,060‐1AP).

### 4.6 Immunofluorescence‐Fluorescent in Situ Hybridization (IF‐FISH)

BJ cells grown on coverslips were transfected with constructs for METTL3/PRKN knockdown or overexpression. After 72 h, IF‐FISH was performed as previously described (Mao et al. 2016), with the following modifications: Cells were incubated with a primary antibody against 53BP1(1:100, NB100‐304, Novus Biologicals) for 1.5 h at room temperature; followed by incubation with Alexa Fluor 488/633 conjugated anti‐rabbit IgG sencondary antibody for 1h at room temperature. After antibody incubation, telomeres were denatured at 85°C and hybridized with Cy3‐labeled PNA telomeric G probes overnight at 37°C. Following hybridization, cells were washed counterstained with DAPI and mounted. Imaging was performed using a Zeiss microscope.

### 4.7 Fluorescent in Situ Hybridization (FISH)

BJ cells were transfected with METTL3 or control siRNA for 2 weeks, following treated with 0.5 μg/mL Nocodazole for 15 h to enrich for metaphase cells. These cells were collected and then hypotonic treatment using 75 mM KCl solution for 30 min at 37°C, followed by fixation with methanol: acetic acid (3:1) twice. Fixed cells were spread onto clean cold slides and digested with 0.5 mg/mL RNase A for 10 min at 37°C. Telomeres were denatured at 85°C and hybridized with FITC‐labeled PNA telomeric C probes. Chromosome were counterstained with DAPI. Imaging was perform using a Nikon microscope.

### 4.8 Quantitative Real‐Time PCR

Total RNA was extracted from cells using the Trizol reagent (Invitrogen) according to the manufacturer's instructions. For RT‐qPCR analysis, cDNA was synthesized from the extracted RNA using HiScript III RT SuperMix for qPCR (+gDNA wiper) (R323‐01, Vazyme) following the manufacturer's protocol. The synthesized cDNA was then analyzed by qPCR using the 2 × RealStar Green Fast Mixture (A311‐10, GenStar). The PCR primer sequences used for the amplification are provided in Table 1.

**TABLE 1:** RT‐qPCR primers.

Primer	Forward sequence (5′‐to −3′)	Reverse sequence (5′‐to −3′)
P16	GGAGCAGCATGGAGCCTTC	CATCATCATGACCTGGTCG
P21	TCACTGTCTTGTACCCTTGTGC	GGCGTTTGGAGTGGTAGAAA
METTL3	CAAGCTGCACTTCAGACGAA	GCTTGGCGTGTGGTCTTT
METTL14	AGAAACTTGCAGGGCTTCCT	TCTTCTTCATATGGCAAATTTTCTT
WTAP	GGCGAAGTGTCGAATGCT	CCAACTGCTGGCGTGTCT
TRF1	AACAGCGCAGAGGCTATTATTC	CCAAGGGTGTAATTCGTTCATCA
TRF2	GTACGGGGACTTCAGACAGAT	CGCGACAGACACT GCATAAC
POT1	CAGCCAATGCAGTATTTTGACC	GGTGCCATCCC ATACCTTTAGAA
BRAD1	GAGCCTGTGTTTAGGAGGA	ACTTCGAGGGCTAAACCACA
KDM1B	GGAACCGTCTTTTTCGCTGG	TTCCCCATCTGGGGTACAGA
MID1	CATCATCGACAGGTTCCAGA	ACAGGTCTTCACAGCGTCCT
BMI1	CGTGTATTGTTCGTTACCTGGA	TTCAGTAGTGGTCTGGTCTTGT
PRKN	AAATGCCCAGACAAGATGCC	GGCCTCTCACGACTGAGTT
SALL1	CAACGTCATCATCGAGAACCTC	AGAGCTAGGAGTTGTTCCATGAG
SYVN1	CTTCGTCAGCCACGCTTATC	CCACGGAGTGCAGCACATAC
18 s RNA	ACGGACCAGAGCGAAAGCAT	GGACATCTAAGGGCATCACAGAC

### 4.9 Quantitative PCR for Telomere Length Detection

BJ cells were cultured in 10 cm dishes, knocking down with siRNA of METTL3 or NC for 1 month. Total DNA extracted from the cells using the HiPure Blood DNA Mini Kit (D3111, Magen) according to the manufacturer's instructions. For the quantitative PCR analysis, 50 ng of the extracted DNA was used. then analyze was performed using the 2 × RealStar Green Fast Mixture (A311‐10, GenStar) with telomere‐specific primers. Albumin, a single‐copy gene, was used as the reference gene. The reaction concentration of DNA and primers were maintained at 2 ng/μL and 10 μM respectively. And the special qPCR procedures refer to published article (Hsieh et al. 2016). The specific PCR primer sequences were provided in the Table 2.

**TABLE 2:** Primers for qPCR‐based telomere length measurement.

Primer	Forward sequence (5′‐to −3′)	Reverse sequence (5′‐to −3′)
Telomere	ACACTAAGGTTTGGGTTTGG GTTTGGGTTTGGGTTAGTGT	TGTTAGGTATCCCTATCCCTA TCCCTATCCCTATCCCTAACA
Albumin	GCTGGGCGGAAATGCTGCAC AGAATCCTTG	TCCCGCCGGAAAAGCATGGT CGCCTGTT

### 4.10 Senescence‐Associated‐β‐Galactosidase (SA‐β‐Gal) Staining

Cells were cultured in 6‐well plates and subjected to knocking down or overexpressing METTL3/PRKN for 1 week. For inducing stress‐induced premature senescence, cells were exposed to X‐ray irradiation at 10 Gy. Immediately after irradiation, the medium was changed, and cells were cultured for an additional week. Subsequently, SA‐β‐gal staining was performed using the senescence β‐Galactosidase Staining Kit (Beyotime) according to the manufacturer's instructions. Briefly, cultured cells were washed with PBS and fixed at room temperature for 15 min. Fixed cells were stained with SA‐β‐gal staining solution at 37°C overnight, Images were captured using a Zeiss microscope.

### 4.11 Immunohistochemical Analysis

Lung tissues were fixed in 4% paraformaldehyde at room temperature overnight, then transferred to 70% ethanol and subsequently embedded in paraffin. Sections of 4 μm thickness were prepared on slides, dewaxed in xylene, and rehydrated (100%, 95% and 70%) followed by PBS buffer. Histopathological analysis was performed on these paraffin‐embedded lung sections stained using Masson's trichrome (MXB Biotechnologies) according to using standard procedures.

For immunohistochemistry, sections were blocked with 5% goat serum in PBS buffer and then incubated overnight at 4°C with primary antibodies. The primary antibodies used included anti‐p21 (1:1000, ab188224, Abcam) and anti‐PRKN (1:100, 14,060‐1AP). After incubation, sections were washed three times with PBS and then incubated with an HRP‐conjugated anti‐rabbit IgG secondary antibody (KPL Inc). Following secondary antibody application, sections were washed again three times with PBS. The detection was carried out using DAB (diaminobenzidine) for enzyme activity visualization and hematoxylin for DNA staining, following established protocols.

The Immunohistochemistry samples are acquired from Canfeng zhang et al. with permission from *EMBO Journal* (Zhang et al. 2024).

### 4.12 Statistical Analysis

Data are presented as mean ± standard error of mean (SEM). All analyses were performed using GraphPad Prism (Version 8.0.1). Normality was assessed per experimental group via Shapiro–Wilk tests (*α* = 0.05), with variance homogeneity evaluated by Brown‐Forsythe tests (*α* = 0.05). For two‐group comparisons, two‐tailed unpaired Student's t‐tests were applied when both assumptions were satisfied; Welch's t‐tests were utilized when normality held but variances differed significantly; Mann–Whitney U tests were employed for non‐normal distributed data. In multi‐group comparisons, one‐way ANOVA with Holm‐Sidak's post hoc testing was conducted only when both normality and variance homogeneity assumptions were met. When either assumption was violated, non‐parametric analysis was performed using Kruskal‐Wallis tests followed by Dunn's post hoc correction with family‐wise error rate control.

## Author Contributions

L. Chen, C. Zhang and Y. Ge designed the study, performed most of the experiments, analyzed the data and wrote the paper. H. Zhou, W. Wei, S. Yang, K. Xiao, G. Huang, X. Li, J. Wang and J. Zheng provided technical assistance. S. Wu, Z. Ju, and Q. Zhou offered ideas and helped analyze the data. S. Wu, Z. Ju and R. Gu. supervised the project.

## Funding

This work was supported by the National Natural Science Foundation of China [32300621, 82230047, 82271590, 82201725, and 32200604], the National Key R&D Program of China [2024YFA0918701], Guangxi Natural Science Foundation [2024GXNSFBA010327 and 2023GXNSFBA026015], the Research Startup Foundation of Nanning First People's Hospital [YN2025003], the innovation team project of universities in Guangdong province [2023KCXTD004].

## Conflicts of Interest

The authors declare no conflicts of interest.

## Supporting information


**Figure S1:** PRKN protein expression is elevated in aged and IPF lung tissues.

## Data Availability

The data supporting the findings of this study are available from the corresponding authors upon request.

## References

[acel70347-bib-0001] Alvarez, D. , N. Cardenes , J. Sellares , et al. 2017. “IPF Lung Fibroblasts Have a Senescent Phenotype.” American Journal of Physiology. Lung Cellular and Molecular Physiology 313: L1164–L1173. 28860144 10.1152/ajplung.00220.2017 PMC6148001

[acel70347-bib-0002] Araya, J. , K. Tsubouchi , N. Sato , et al. 2019. “PRKN‐Regulated Mitophagy and Cellular Senescence During COPD Pathogenesis.” Autophagy 15: 510–526. 30290714 10.1080/15548627.2018.1532259 PMC6351145

[acel70347-bib-0003] Arcidiacono, O. A. , J. Krejci , and E. Bartova . 2020. “The Distinct Function and Localization of METTL3/METTL14 and METTL16 Enzymes in Cardiomyocytes.” International Journal of Molecular Sciences 21: 8139. 33143367 10.3390/ijms21218139 PMC7663386

[acel70347-bib-0004] Chen, L. , C. Zhang , W. Ma , J. Huang , Y. Zhao , and H. Liu . 2022. “METTL3‐Mediated m6A Modification Stabilizes TERRA and Maintains Telomere Stability.” Nucleic Acids Research 50: 11619–11634. 36399511 10.1093/nar/gkac1027 PMC9723618

[acel70347-bib-0005] d'Adda di Fagagna, F. 2008. “Living on a Break: Cellular Senescence as a DNA‐Damage Response.” Nature Reviews. Cancer 8: 512–522. 18574463 10.1038/nrc2440

[acel70347-bib-0006] Du, Y. , G. Hou , H. Zhang , et al. 2018. “SUMOylation of the m6A‐RNA Methyltransferase METTL3 Modulates Its Function.” Nucleic Acids Research 46: 5195–5208. 29506078 10.1093/nar/gky156 PMC6007514

[acel70347-bib-0007] Hershko, A. , and A. Ciechanover . 1998. “The Ubiquitin System.” Annual Review of Biochemistry 67: 425–479. 10.1146/annurev.biochem.67.1.425 9759494

[acel70347-bib-0008] Hsieh, A. Y. Y. , S. Saberi , A. Ajaykumar , et al. 2016. “Optimization of a Relative Telomere Length Assay by3 Monochromatic Multiplex Real‐Time Quantitative PCR on the LightCycler 480: Sources of Variability and Quality Control Considerations.” Journal of Molecular Diagnostics 18: 425–437. 10.1016/j.jmoldx.2016.01.004 PMC5818633 26972047

[acel70347-bib-0009] Ito, S. , J. Araya , Y. Kurita , et al. 2015. “PARK2‐Mediated Mitophagy Is Involved in Regulation of HBEC Senescence in COPD Pathogenesis.” Autophagy 11: 547–559. 25714760 10.1080/15548627.2015.1017190 PMC4502689

[acel70347-bib-0010] Jin, X. , K. Wang , L. Wang , et al. 2022. “RAB7 Activity Is Required for the Regulation of Mitophagy in Oocyte Meiosis and Oocyte Quality Control During Ovarian Aging.” Autophagy 18: 643–660. 34229552 10.1080/15548627.2021.1946739 PMC9037413

[acel70347-bib-0011] Jing, H. , J. Song , J. Sun , et al. 2024. “METTL3 Governs Thymocyte Development and Thymic Involution by Regulating Ferroptosis.” Nature Aging 4: 1813–1827. 39443728 10.1038/s43587-024-00724-x

[acel70347-bib-0012] Lee, J. H. , J. Hong , Z. Zhang , et al. 2021. “Regulation of Telomere Homeostasis and Genomic Stability in Cancer by N (6)‐adenosine Methylation (m(6)A).” Science Advances 7: eabg7073. 34321211 10.1126/sciadv.abg7073 PMC8318370

[acel70347-bib-0013] Liao, L. , Y. He , S. J. Li , et al. 2022. “Anti‐HIV Drug Elvitegravir Suppresses Cancer Metastasis via Increased Proteasomal Degradation of m6A Methyltransferase METTL3.” Cancer Research 82: 2444–2457. 35507004 10.1158/0008-5472.CAN-21-4124

[acel70347-bib-0014] Liu, S. , Q. Li , K. Chen , et al. 2020. “The Emerging Molecular Mechanism of m(6)A Modulators in Tumorigenesis and Cancer Progression.” Biomedicine & pharmacotherapy = Biomedecine & pharmacotherapie 127: 110098. 32299028 10.1016/j.biopha.2020.110098

[acel70347-bib-0015] Mao, P. , J. Liu , Z. Zhang , et al. 2016. “Homologous Recombination‐Dependent Repair of Telomeric DSBs in Proliferating Human Cells.” Nature Communications 7: 12154. 10.1038/ncomms12154 PMC4942568 27396625

[acel70347-bib-0016] Mao, W. , Q. Jiang , Y. Feng , et al. 2025. “TRIM21‐Mediated METTL3 Degradation Promotes PDAC Ferroptosis and Enhances the Efficacy of Anti‐PD‐1 Immunotherapy.” Cell Death & Disease 16: 240. 40175350 10.1038/s41419-025-07550-y PMC11965403

[acel70347-bib-0017] Meyer, K. D. , and S. R. Jaffrey . 2014. “The Dynamic Epitranscriptome: N6‐Methyladenosine and Gene Expression Control.” Nature Reviews. Molecular Cell Biology 15: 313–326. 24713629 10.1038/nrm3785 PMC4393108

[acel70347-bib-0018] Min, K. W. , R. W. Zealy , S. Davila , et al. 2018. “Profiling of m6A RNA Modifications Identified an Age‐Associated Regulation of AGO2 mRNA Stability.” Aging Cell 17: e12753. 29573145 10.1111/acel.12753 PMC5946072

[acel70347-bib-0019] Munoz‐Espin, D. , and M. Serrano . 2014. “Cellular Senescence: From Physiology to Pathology.” Nature Reviews. Molecular Cell Biology 15: 482–496. 24954210 10.1038/nrm3823

[acel70347-bib-0020] Ovadya, Y. , T. Landsberger , H. Leins , et al. 2018. “Impaired Immune Surveillance Accelerates Accumulation of Senescent Cells and Aging.” Nature Communications 9: 5435. 10.1038/s41467-018-07825-3 PMC6303397 30575733

[acel70347-bib-0021] Raghuram, G. V. , and P. K. Mishra . 2014. “Stress Induced Premature Senescence: A New Culprit in Ovarian Tumorigenesis?” Indian Journal of Medical Research 140: S120–S129. 25673532 PMC4345742

[acel70347-bib-0022] Roundtree, I. A. , M. E. Evans , T. Pan , and C. He . 2017. “Dynamic RNA Modifications in Gene Expression Regulation.” Cell 169: 1187–1200. 28622506 10.1016/j.cell.2017.05.045 PMC5657247

[acel70347-bib-0023] Schafer, M. J. , T. A. White , K. Iijima , et al. 2017. “Cellular Senescence Mediates Fibrotic Pulmonary Disease.” Nature Communications 8: 14532. 10.1038/ncomms14532 PMC5331226 28230051

[acel70347-bib-0024] Shi, H. , J. Wei , and C. He . 2019. “Where, When, and How: Context‐Dependent Functions of RNA Methylation Writers, Readers, and Erasers.” Molecular Cell 74: 640–650. 31100245 10.1016/j.molcel.2019.04.025 PMC6527355

[acel70347-bib-0025] Wahabi, K. , A. Perwez , and M. A. Rizvi . 2018. “Parkin in Parkinson's Disease and Cancer: A Double‐Edged Sword.” Molecular Neurobiology 55: 6788–6800. 29349575 10.1007/s12035-018-0879-1

[acel70347-bib-0026] Wang, C. , D. Jurk , M. Maddick , G. Nelson , C. Martin‐Ruiz , and T. von Zglinicki . 2009. “DNA Damage Response and Cellular Senescence in Tissues of Aging Mice.” Aging Cell 8: 311–323. 19627270 10.1111/j.1474-9726.2009.00481.x

[acel70347-bib-0027] Wang, X. , Y. Li , M. He , et al. 2022. “UbiBrowser 2.0: A Comprehensive Resource for Proteome‐Wide Known and Predicted Ubiquitin Ligase/Deubiquitinase‐Substrate Interactions in Eukaryotic Species.” Nucleic Acids Research 50: D719–D728. 34669962 10.1093/nar/gkab962 PMC8728189

[acel70347-bib-0028] Wang, Y. , Y. Chen , H. Xiao , et al. 2024. “METTL3‐Mediated m6A Modification Increases Hspa1a Stability to Inhibit Osteoblast Aging.” Cell Death Discov 10: 155. 38538596 10.1038/s41420-024-01925-4 PMC10973419

[acel70347-bib-0029] Wu, Z. , M. Lu , D. Liu , et al. 2023. “M(6)A Epitranscriptomic Regulation of Tissue Homeostasis During Primate Aging.” Nature Aging 3: 705–721. 37118553 10.1038/s43587-023-00393-2

[acel70347-bib-0030] Wu, Z. , Y. Shi , M. Lu , et al. 2020. “METTL3 Counteracts Premature Aging via m6A‐Dependent Stabilization of MIS12 mRNA.” Nucleic Acids Research 48: 11083–11096. 33035345 10.1093/nar/gkaa816 PMC7641765

[acel70347-bib-0031] Yang, X. , F. Han , X. Hu , et al. 2023. “EIF4A3‐Induced Circ_0001187 Facilitates AML Suppression Through Promoting Ubiquitin‐Proteasomal Degradation of METTL3 and Decreasing m6A Modification Level Mediated by miR‐499a‐5p/RNF113A Pathway.” Biomarker Research 11: 59. 37280654 10.1186/s40364-023-00495-4 PMC10243067

[acel70347-bib-0032] Zhang, C. , L. Chen , D. Peng , et al. 2020. “METTL3 and N6‐Methyladenosine Promote Homologous Recombination‐Mediated Repair of DSBs by Modulating DNA‐RNA Hybrid Accumulation.” Molecular Cell 79: 425–442. 32615088 10.1016/j.molcel.2020.06.017

[acel70347-bib-0033] Zhang, C. , L. Chen , C. Xie , et al. 2024. “YTHDC1 Delays Cellular Senescence and Pulmonary Fibrosis by Activating ATR in an m6A‐Independent Manner.” EMBO Journal 43: 61–86. 38177310 10.1038/s44318-023-00003-2 PMC10883269

