# Supplementary material for: PRKN‐Mediated Ubiquitin‐Proteasome Degradation of METTL3 Promotes Cellular Senescence
Source: Aging Cell. 2025 Dec 29;25(1):e70347. doi: 10.1111/acel.70347 (PMC12745842; doi:10.1111/acel.70347)
Supplement: Supplementary file 1 — Figure S1: PRKN protein expression is elevated in aged and IPF lung tissues. [file ACEL-25-e70347-s001.pdf]

Fig.S1

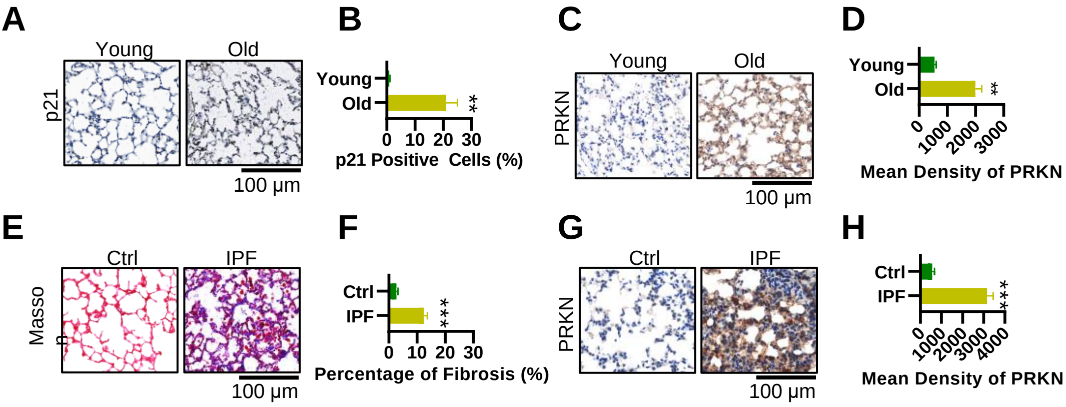

Figure S1. PRKN Protein Expression Is Elevated in Aged and IPF Lung Tissues. (A) Representative images of p21 immunohistochemistry (IHC) from lung tissue of young and old mice. Scale bar:100 μm. (B) Quantification of p21 signal from panel (A). (C) Representative images of PRKN IHC from lung tissue of young and old mice. Scale bar:100 μm. (D) Quantification of PRKN signal from panel (C). (E) Masson's trichrome staining was used to assess fibrosis levels of fibrosis from control and idiopathic pulmonary fibrosis (IPF) model mice. (F) Quantification of masson signal in panel (E). (G) Representative images of PRKN IHC are shown. Scale bar:100 μm. (H) Quantification of PRKN signal in panel (G). Data represent mean ± SEM of three or more biological replicates. Statistical significance was assessed using two-tailed unpaired Student's t-tests (for data with equal variances) or Welch's corrected t-tests (for unequal variances): $**p < 0.01$, $***p < 0.001$.
